# Thermoelectric performance enhancement of eco-friendly Cu_2_Se through incorporating CB_4_

**DOI:** 10.1039/d2ra01546c

**Published:** 2022-05-11

**Authors:** Wen Xie, Feng Liu, Yingxiang Zheng, Nina Ge, Bo Dai, Xiaowei Zhang

**Affiliations:** State Key Laboratory of Environment-Friendly Energy Materials, Southwest University of Science and Technology Mianyang 621010 China hedge80@sina.com.cn +86-0816-2419-492 +86-0816-2419-492

## Abstract

We have prepared Cu_2_Se + *x* wt% CB_4_ composites with *x* = 0, 0.1, 0.3, 0.5, and 0.7 by a hydrothermal method and hot-pressing technique. The structural and compositional analysis indicates that pure phase Cu_2_Se powders were synthesized and the densified layered bulk samples were obtained. Electrical properties testing showed that the sample with *x* = 0.5 has the high power factor of 0.886 mW m^−1^ K^−2^ due to its high Seebeck coefficient. Meanwhile, the thermal conductivity was suppressed to 0.6 W m^−1^ K^−1^ at 773 K. As a result, the final optimized *ZT* value of 1.46 at 773 K was achieved. These results suggest that CB_4_ could be an alternative inclusion to improve effectively the thermoelectric performance of Cu_2_Se.

## Abstract 2

Thermoelectric performance enhancement in a liquid-like material Cu_2_Se by introducing CB_4_ nanopowders based on the hydrothermal method and hot-pressing technique.

## Graphical abstract

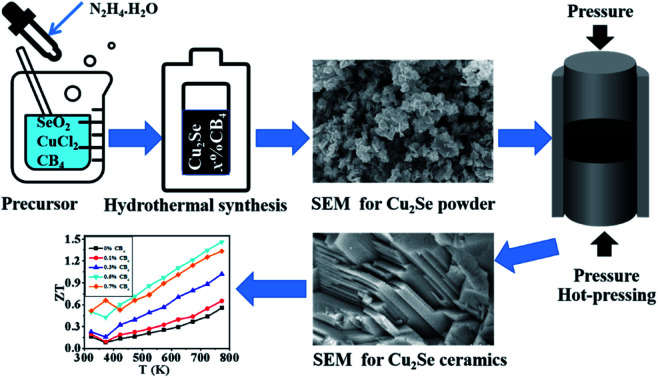

## 1. Introduction

With the wide demand for environment-friendly energy, thermoelectric (TE) materials have received significant attention during the past two decades.^1–6^ TE materials can realize the conversion directly between thermal and electrical energy.^7–9^ High-efficiency TE materials require a high dimensionless figure of merit (*ZT*), *ZT* = *S*^2^*σT*/*κ*, where *S* is the Seebeck coefficient, *σ* is the electrical conductivity, *T* is the absolute temperature, and *κ* is the total thermal conductivity respectively.^10–13^ Numerous exploratory efforts tried to optimize these parameters, *i.e.*, enhance the power factor (PF = *S*^2^*σ*) and reduce the thermal conductivity simultaneously, to achieve high TE performance.^14,15^ However, these parameters are strongly coupled together, making it challenging to improve the *ZT*s.^16,17^

Chalcogenide TE materials have attracted widespread interests and have been intensively studied because of their intrinsically low lattice thermal conductivity (*κ*_L_).^18–21^ Very recently, Chung and co-workers reported^22^ the ultralow *κ*_L_ of 0.07 W m^−1^ K^−1^ obtained in polycrystal SnSe and thereby got a high *ZT* of 3.1, which breaks thermoelectric performance limits.^23^ Cu-based chalcogenide also received much attention for environmental-friendly TE application and Cu_2_Se is one of the strongest candidates.^24,25^ To improve the TE performance of Cu_2_Se, numerous strategies have been developed, such as nano-engineering,^26,27^ solid solution alloying,^28,29^ element doping,^30–32^ and introducing highly dispersed nanoparticles.^33–36^ Among them, adding nanoparticles to Cu_2_Se matrix has been demonstrated to be an effective and prevailing approach to improve TE performance. The nanoparticles in composites, such as TiO_2_,^37^ graphene,^38^ nano-boron,^39^ carbon dots^40,41^ and CNTs,^42^ can effectively scatter intermediate frequency phonons and hence suppress *κ*_L_.

In this work, we try to introduce boron carbide (CB_4_) as a second phase in the Cu_2_Se matrix because nano-CB_4_ has good thermal stability, high temperature electrical conductivity and relatively large Seebeck coefficient.^43^ For preparing Cu_2_Se samples with different contents of CB_4_, the hydrothermal method and hot-pressing technique are employed. The results show that CB_4_ inclusions do reduce *κ* significantly and meanwhile enhance PF slightly over a wide range of temperature. As a result, the highest *ZT* reaches 1.46 at 773 K, which is much higher than the value of undoped sample.

## 2. Experimental section

Cu_2_Se nanopowders were prepared by hydrothermal method and CB_4_ nanopowders were added during the hydrothermal process. We first mixed 20 mmol copper chloride dehydrate (CuCl_2_·2H_2_O, 99.9%), 10 mmol selenium dioxide (SeO_2_, 99.9%) and a recommended amount of CB_4_ (99%) into about 60 milliliters deionized water, and then magnetic stirred continuously for 12 h. The amount of CB_4_ is determined by the mass ratio of Cu_2_Se and CB_4_. In this work, we consider five Cu_2_Se + *x* wt% CB_4_ (*x* = 0, 0.1, 0.3, 0.5, 0.7) samples, which are named as S0, S1, S3, S5 and S7 respectively for the convenience of description. After the components were completely dissolved, we added 20 milliliters hydrazine hydrate (N_2_H_4_·H_2_O) into the mixture solution. The solution was then loaded into a Teflon-lined stainless steel autoclave and sealed. The autoclave was heated at 453 K for 24 h and cooled down to room temperature naturally. The product was collected by centrifuging and washed by deionized water and anhydrous ethanol for several times. We finally dried the product in vacuum at 333 K for at least 12 h. For getting the final samples, the synthesized powders were sintered into pallets with a diameter of 20 mm by hot-pressing method at 973 K and under a pressure of 40 MPa in the vacuum for 30 minutes.

The phase compositions of the prepared samples were identified by X-ray diffraction measurements (XRD, PANalytical, Netherlands) with Cu Kα radiation. The fracture surface microstructure was observed by field emission scanning electron microscopy (SEM, TM4000) and Energy-dispersive X-ray spectroscopy (EDS, X-MAX^N^20, England). Seebeck coefficients and electrical conductivity simultaneously were measured by using a CTA-3 system (Cryoall, China) from 323 K to 773 K in a helium atmosphere. *κ* was calculated according to the formula *κ* = *ρDC*_p_, where *ρ* is the density of the samples, *D* is the thermal diffusivity, and *C*_p_ is the heat capacity respectively. *D* was measured using the laser flash method (DFL-1600, Netzsch) on a square piece specimen with 10 mm in side and 1.5 mm in thickness. *C*_p_ was measured by differential scanning calorimetry (DSCQ-2000, TA). The density of the samples *ρ* was obtained from Archimedes' method.

## 3. Results and discussion

**Fig. 1:**
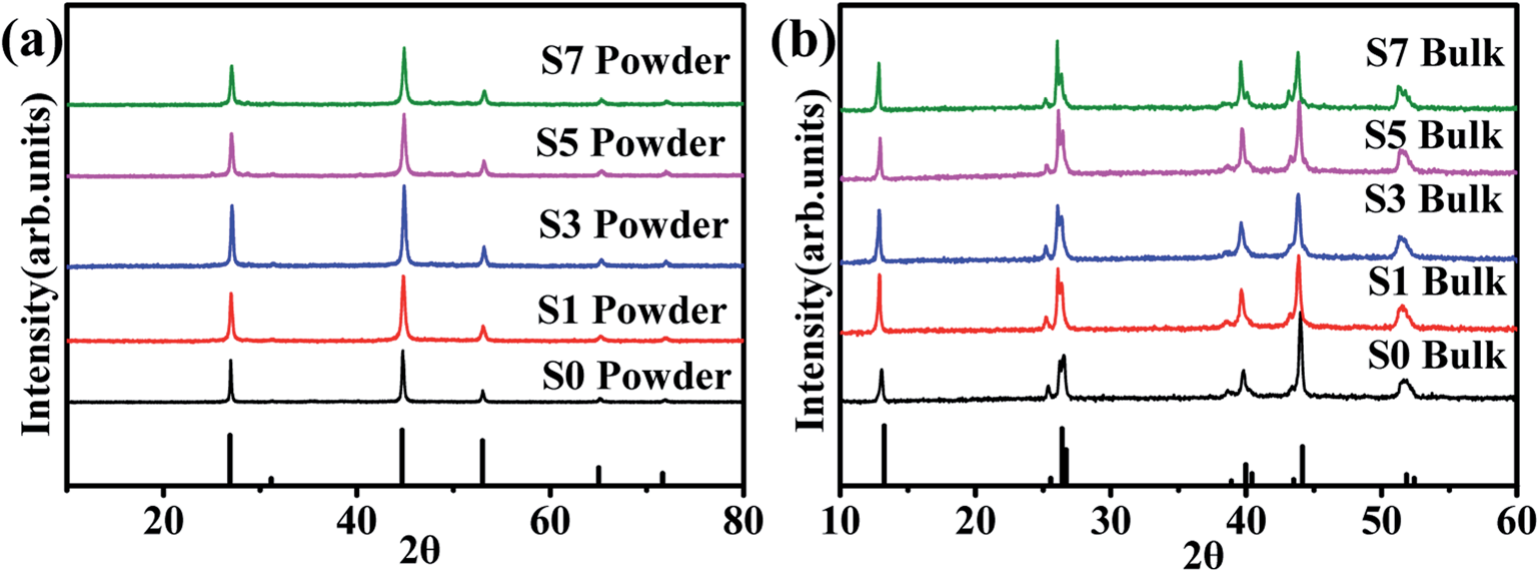
(a) XRD patterns of the β-Cu_2_Se (ICDD file No. 00-006-0680) synthesized nanopowders. (b) XRD patterns of α-Cu_2_Se (ICDD file No. 00-019-0401) bulk samples.

Fig. 1(a) shows the XRD patterns of the synthesized Cu_2_Se + *x* wt% CB_4_ (*x* = 0, 0.1, 0.3, 0.5, 0.7) nanopowders. The main diffraction peaks are all in consistent with the ICDD file No. 00-006-0680, indicating β-phase Cu_2_Se is prepared successfully.^40,44–46^ No CB_4_ phase is to be detected, which might be due to introducing only a small amount into the main β-phase Cu_2_Se. Fig. 1(b) shows the XRD patterns of the sintered bulk samples. The results match with the ICDD file No. 00-019-0401 very well, indicating that the as-prepared β-Cu_2_Se nanostructures has transferred to pure α-Cu_2_Se after sintering.^40,46,47^

**Fig. 2:**
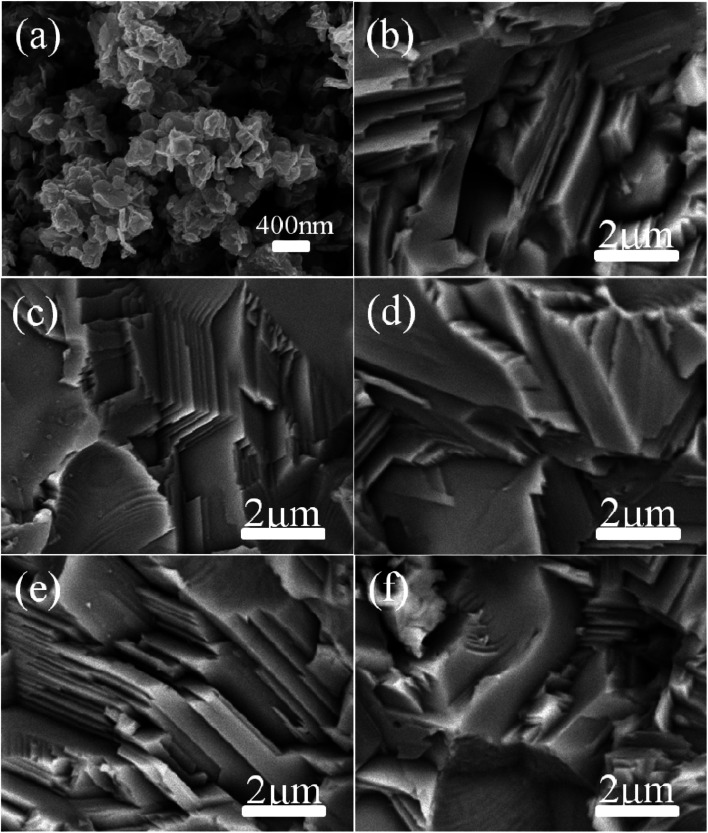
SEM images for (a) Cu_2_Se nanopowders, the samples of (b) S0, (c) S1, (d) S3, (e) S5 and (f) S7.

**Fig. 3:**
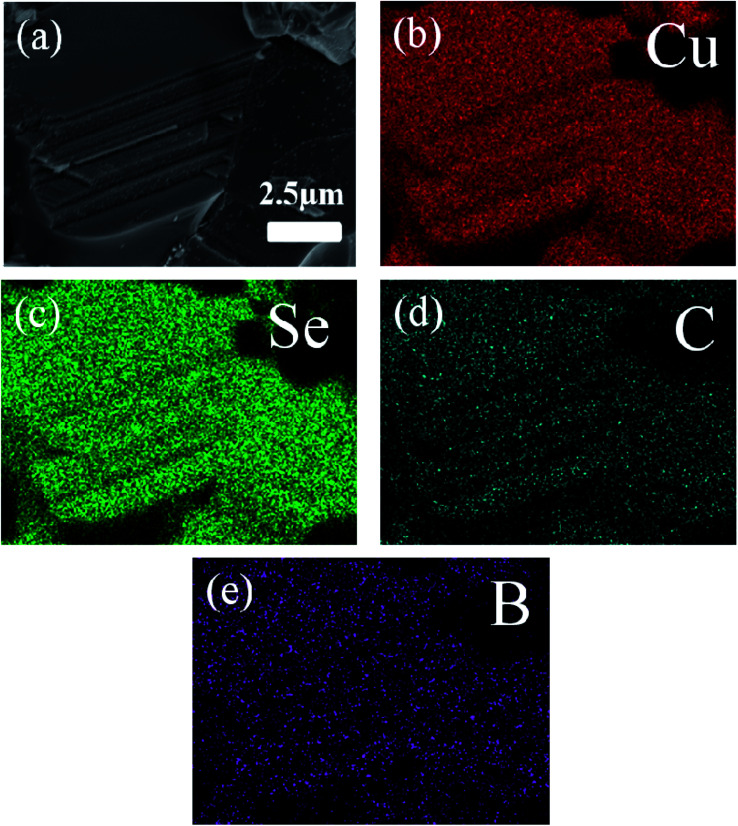
(a) SEM image of the typical morphology of the S5. (b)–(e) The corresponding EDS maps.

The typical morphology of the synthesized Cu_2_Se precursor nanopowders is shown in Fig. 2(a). Nanoplates with a lateral size of 100–500 nm, often aggregating together, can be clearly observed. Fig. 2(b–f) display the cross-sectional microstructures of the hot-pressed Cu_2_Se + *x* wt% CB_4_ bulk samples. Similar lamellar structure is observed for each sample. Such structure is conducive to reducing the thermal conductivity due to the increased scattering of phonons between layered interfaces.^48^ A few pores on the surface can be spotted which might be due to Se volatilization during the sintering process and hence the corresponding density is slightly lower than the pure Cu_2_Se. The samples S0, S1, S3, S5, and S7 have densities of 5.75, 5.65, 5.38, 5.29, 5.20 g cm^−3^ respectively (theoretical density of Cu_2_Se is 6.74 g cm^−3^). Fig. 3 shows the surface EDS mapping of the sample S5. These maps suggest that copper, selenium, carbon and boron signals overlap, showing CB_4_ is uniformly distributed in the sample.

**Fig. 4:**
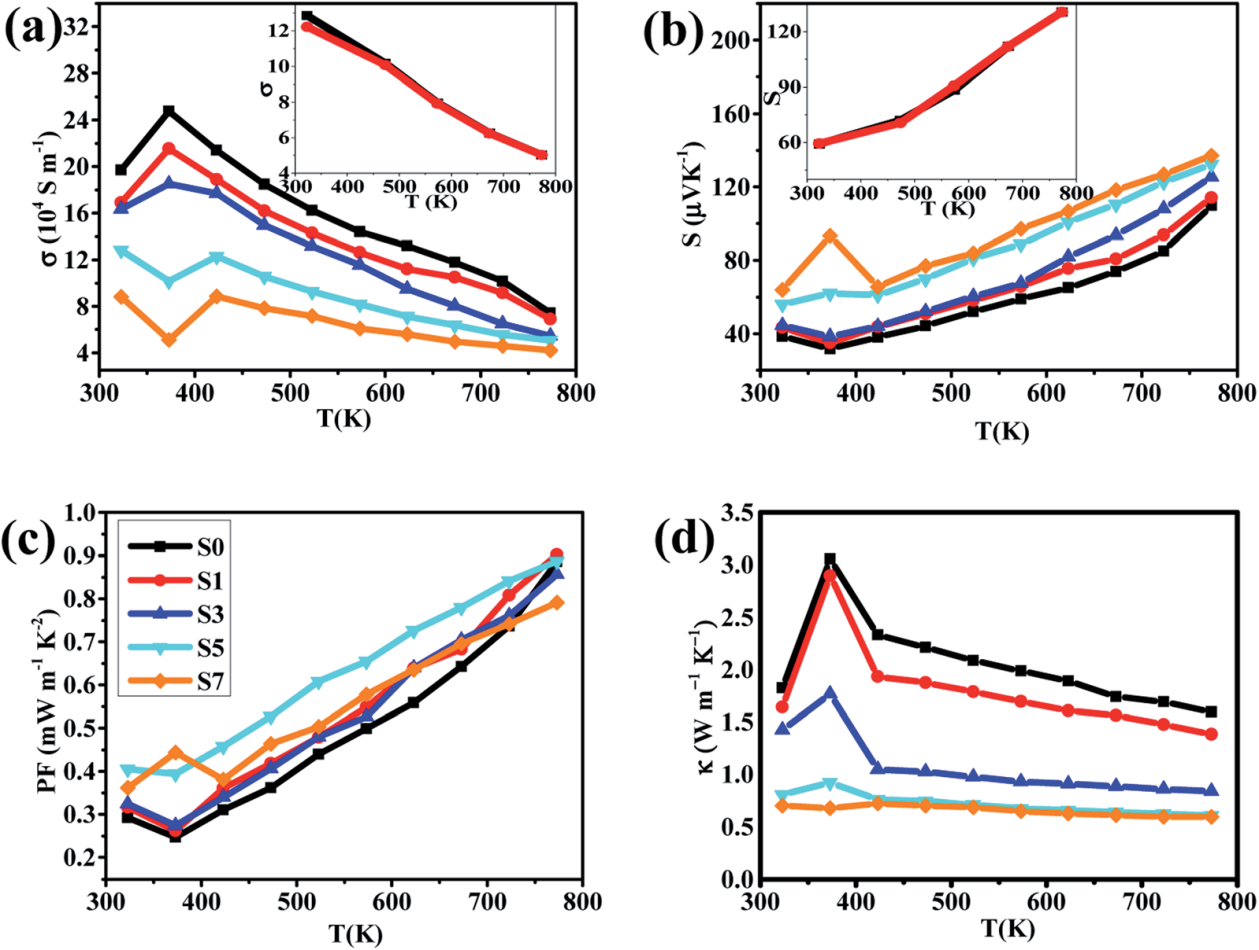
Temperature dependence of (a) electrical conductivities, (b) Seebeck coefficients, (c) power factors and (d) thermal conductivities for the samples of S0, S1, S3, S5 and S7. Insets of (a) and (b) are the heating (red line) and cooling (black line) curves for electrical conductivity and Seebeck coefficient of sample S5 respectively.

The temperature dependence of the electrical conductivity *σ* of our samples is shown in Fig. 4(a). Within the temperature range of 400–773 K, the electrical conductivity *σ* of all samples decreases monotonously with temperature increasing, which shows the typical behaviors of metallic-like characteristic. However, we can clearly find that the electrical conductivity *σ* varies abnormally around 400 K, which have been proved to be due to the phase transition from α-Cu_2_Se to β-Cu_2_Se.^49^ In addition, the electrical conductivity *σ* decreases with the increasing of CB_4_ components at each testing temperature. For example, compared to the sample S0, the electrical conductivity *σ* is about 3 times smaller around 400 K and 2 times smaller at 773 K for the sample S7. This trend in electrical conductivity *σ* is very different from the report of Cu_2_Se/SiC composite,^27^ but similar to the report of Cu_2_S/SiC composite.^17^ For clarifying the underlying mechanism of the electrical transport behaviors, the room temperature carrier concentration (*n*) and carrier mobility (*μ*) are measured, as shown in Table 1. Clearly, there is a reduction in carrier concentration and an increase in carrier mobility with increasing of the CB_4_ composite content. For the superionic conductor Cu_2_Se, unstable copper ions show a liquid-like behavior and hence cause the unrepeatable electrical transport properties. The carrier concentration *n* contains two parts: the majority carrier (*n*_hole_) and the ion carrier (*n*_ion_), and therefore, the electrical conductivity *σ* = *σ*_hole_ + *σ*_ion_. In our work, the introduction of CB_4_ might effectively block the migration of copper ions, which causes the reduction in *n*_ion_ and hence in the whole carrier concentration *n*. In addition, the migration restrictions of copper ions can also effectively enhance the stability of Cu_2_Se chemical structure, which is confirmed by the cyclic testing shown in Fig. 4. The increase in mobility *μ* might be due to the grain growth during the process of hot pressing, as can be clearly seen in Fig. 2. Because of the difficulty for measuring the transport of ions, more related discussions do not be present here.

**Table 1:** Carrier concentration (*n*), mobility (*μ*) at room temperature

Sample	*n*_hole_ + *n*_ion_ (10^19^ cm^−3^)	*μ* (cm^2^ V^−1^ s^−1^)
S0	7.9	73.6
S1	6.6	101
S3	5.5	104
S5	5.3	112
S7	3.6	119

In our work, the samples S5 and S7 show a quite low electrical conductivity *σ*. This fact would not be very good for optimizing the thermoelectric properties of Cu_2_Se. However, thankfully, the reduction in electrical conductivity *σ* is often accompanied by enhancement of the Seebeck coefficient. As we can see from Fig. 4(b), the samples S5 and S7 exhibit a larger Seebeck coefficient than the others within the entire testing temperature range and the optimal value reaches 137 μV K^−1^ at 773 K for the sample S7. Additionally, the positive Seebeck coefficient for all samples suggests the *p*-type electrical transport in Cu_2_Se. The calculated PFs of the samples are shown in Fig. 4(c). Very clearly, PFs curves of the samples S0, S1, S3 and S5 show a valley around 400 K due to the phase transition from β-Cu_2_Se to α-Cu_2_Se,^50,51^ while the sample S7 has the abnormal high PF because of its very high *S* and low *σ*. However, we cannot evaluate precisely these parameters around 400 K because they are very sensitive to temperature change during the process of phase transformation. This might be the reason that some previous studies did not show the related data close to 400 K.

As *T* >400 K, all PFs increase rapidly as temperature increases. However, the sample S7 does not show the superior PF value, which might be due to its very low electrical conductivity. In contrast, the sample S5 has the optimal PF as *T* >400 K (PF_max_ = 0.886 mW m^−1^ K^−2^ at 773 K), indicating that a moderate content of CB_4_ composites may result in a better electrical performance of Cu_2_Se.

**Fig. 5:**
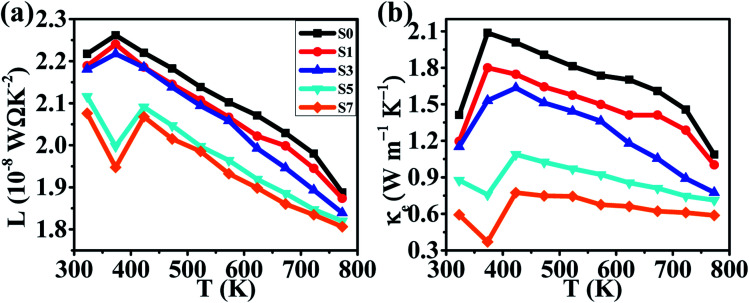
Temperature dependence of (a) the calculated Lorenz number, (b) the calculated thermal conductivities for samples of S0, S1, S3, S5 and S7.

Another important parameter to determine the TE properties is the thermal conductivity *κ*. The thermal conductivity can be divided into two parts: *κ* = *κ*_L_ + *κ*_carrier_, where *κ*_L_ comes from heat phonons travelling through the crystal lattice and *κ*_carrier_ arises from heat carriers' movement. Generally, at high temperature, *κ*_L_ dominates in Cu_2_Se matrix.^39^ The measured thermal conductivity is shown in Fig. 4(d). Clearly, all of the thermal conductivity *κ* decrease with temperature increasing, which is a reasonable trend because of the increasing in phonon scattering and the reduction in carrier concentration. For a fixed temperature, as CB_4_ component increases from 0.0 to 0.7%, the thermal conductivity decreases very rapidly. To take a step further, carrier thermal conductivity (*κ*_carrier_) is calculated by using the Wiedemann–Franz relationship, *κ*_carrier_ = *Lσ*T, where *L* is the Lorenz number. When the detailed band structure and scattering mechanism are not known, the Lorenz number can not be viewed as a constant but can be calculated by *L* = 1.5 + exp [−|*S*|/116], where *L* is in unit of 10^−8^ W Ω K^−2^ and *S* (Seebeck coefficient) in unit of μV K−^1^.^52^ The calculated *L* and *κ*_carrier_ are showed in Fig. 5. However, it seems that the measured total thermal conductivity of S5 and S7 are lower than the calculated values. The similar phenomenon happened to Cu_2_Se/SiC system^27^ and Cu–S system.^17,53^ It might be due to the different thermal transport properties between electron/hole carriers and ion carriers. The equation *κ*_carrier_ = *LσT* may not be suitable for Cu_2_Se/CB_4_ composite. The ion carriers have a non-negligible contribution to total thermal transport, so the modified equation can be taken as *κ*_carrier_ = *L* (*σ* − *σ*_ion_)*T* + *κ*_ion_,^27,30,53^ where *κ*_ion_ is the ionic thermal conductivity, *σ*_ion_ is ionic electrical conductivity. However, *σ*_ion_ and *κ*_ion_ cannot be measured precisely now and hence the carrier thermal conductivity and lattice thermal conductivity are not offered here.

**Fig. 6:**
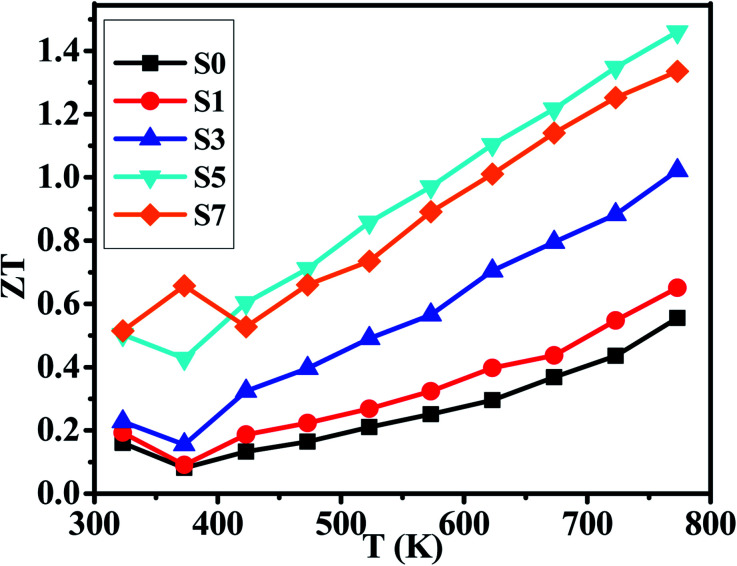
Temperature dependence of *ZT*s for samples of S0, S1, S3, S5 and S7.

Considering the total thermal conductivity, the samples S5 and S7 remain very low values and very close to each other as *T* >400 K. The lowest *κ* value is 0.59 W m^−1^ K^−1^ at 773 K for S7 sample. This result indicates that the higher concentration of CB_4_ inclusions could not improve further the thermal conductivity. Moreover, as shown in Fig. 4(c), the PF of sample S7 is also smaller than sample S5. Therefore, in this work, the S5 sample would show the optimal thermoelectric performance. This can be confirmed positively by the relationship between *ZT* values and temperature, as shown in Fig. 6. Sample S5 exhibits the largest *ZT* than the others as *T* >400 K and the largest value is of 1.46 at 773 K, which is much larger than that of undoped sample S0 (*ZT*_max_ = 0.55 at 773 K). The average *ZT* value of S5 (*ZT*_average_ = 1.034) is also larger than one within the temperature range of 400–773 K. These results suggest that Cu_2_Se compositing a certain amount of CB_4_ is very promising for TE application.

## 4. Conclusions

We have prepared the pure phase Cu_2_Se + *x* wt% CB_4_ (*x* = 0, 0.1, 0.3, 0.5, 0.7) nanopowders by hydrothermal method and have sintered the corresponding bulk samples by hot-pressing technique. With the content of CB_4_ increasing, the electrical conductivities of our samples decrease very rapidly, while the Seebeck coefficients steadily increase. The final PFs show that the sample S5 possesses the optimal value within the testing temperature range. The thermal conductivity testing confirms that the CB_4_ nano-inclusions can drastically reduce the *κ* of our samples. The lowest *κ* value is 0.59 W m^−1^ K^−1^ at 773 K for S7 sample. However, the *κ* of sample S5 is very close to that of sample S7 as *T* >400 K. Finally, the optimized *ZT* value of 1.46 at 773 K is achieved for S5 sample, which is about 165% larger than the undoped sample.

## Conflicts of interest

The authors have no conflicts to declare.

## Supplementary Material
